# Paratyphoid fever and the genomics of *Salmonella enterica* serovar Paratyphi A in Taiwan

**DOI:** 10.1371/journal.pntd.0013048

**Published:** 2025-09-11

**Authors:** Ying-Shu Liao, Yu-Ping Hong, Bo-Han Chen, You-Wun Wan, Ru-Hsiou Teng, Shiu-Yun Liang, Hsiao Lun Wei, Jui-Hsien Chang, Ming-Hao Yang, Chi-Sen Tsao, Chien-Shun Chiou

**Affiliations:** Center for Research, Diagnostics and Vaccine Development, Centers for Disease Control, Ministry of Health and Welfare, Taipei, Taiwan; Mahidol Univ, Fac Trop Med, THAILAND

## Abstract

**Background:**

*Salmonella enterica* serovar Paratyphi A (*S.* Paratyphi A) has emerged as a significant global health concern due to the progressive development of antimicrobial resistance and its broader geographic distribution. In Taiwan, paratyphoid fever was historically rare and predominantly associated with imported cases. Since 2022, however, a marked increase in domestically acquired infections has been observed, prompting investigations into their origin and likely route of introduction.

**Methods:**

We analyzed surveillance data on 223 patients with paratyphoid fever reported in Taiwan between January 2001 and December 2024. Whole-genome sequencing and antimicrobial susceptibility testing were performed on 88 *S.* Paratyphi A isolates obtained from both imported and domestically acquired infections from 2007 to 2024. Phylogenetic analysis and genotyping were conducted to assess genetic relatedness and to trace potential sources of introduction by comparing them with global isolates.

**Results:**

Although 55.2% of paratyphoid fever infections were imported, domestically acquired infections became predominant after 2022. Most isolates (76.1%) were resistant to nalidixic acid and nonsusceptible to ciprofloxacin due to *gyrA* mutations at codon 83 (S83F or S83Y). The majority of domestic isolates were classified as ST129 and paratype 2.4 and showed close genetic relatedness to strains from Indonesia. Of the 31 domestic isolates collected between 2022 and 2024, 30 clustered with Indonesian strains, and 28 exhibited nearly identical genomic profiles, which suggested a prolonged outbreak likely linked to a common external source, such as contaminated imported food.

**Conclusions:**

The genomic evidence suggests that the recent increase in domestically acquired *S.* Paratyphi A infections in Taiwan represents a prolonged outbreak rather than a sustained epidemiological shift. These infections were closely related to strains from Indonesia, suggesting a potential epidemiological link between the two countries in the transmission of paratyphoid fever. While 76.1% of isolates were nonsusceptible to ciprofloxacin due to *gyrA* mutations, susceptibility to traditional first-line agents remained high. The observed decline in case numbers in 2024 may indicate that the outbreak is subsiding. Genomic surveillance played a crucial role in tracing sources of infection and informing targeted public health responses.

## Author summary

Paratyphoid fever, caused by *Salmonella enterica* serovar Paratyphi A (*S.* Paratyphi A), is typically associated with endemic regions in South and Southeast Asia. In Taiwan, the disease has historically been rare and primarily linked to imported cases. However, since 2022, the number of domestically acquired infections has increased. We analyzed 88 *S.* Paratyphi A isolates collected in Taiwan between 2007 and 2024 using whole-genome sequencing and antimicrobial susceptibility testing. While 76.1% of isolates were resistant to nalidixic acid and exhibited reduced susceptibility to ciprofloxacin, these phenotypes were associated with *gyrA* mutations at codon 83, which are known to impair the efficacy of fluoroquinolones. Importantly, all isolates remained susceptible to 13 other tested antimicrobials, including older drugs such as ampicillin, chloramphenicol, and trimethoprim-sulfamethoxazole. Phylogenetic analysis showed that most domestic isolates belonged to the same genetic lineage as strains from Indonesia. Notably, 30 of the 31 isolates collected from domestic cases between 2022 and 2024 clustered with Indonesian strains, and 28 exhibited nearly identical genetic profiles, despite the patients having no epidemiological links. This suggests that the infections likely originated from a common external source, such as contaminated imported food or an environmental reservoir, rather than through sustained local transmission. Our study highlights the value of genomic surveillance in tracing infection sources and provides evidence to support targeted public health interventions and treatment strategies that preserve the use of traditional first-line antibiotics where susceptibility remains high.

## Introduction

Paratyphoid fever is a systemic illness caused by *Salmonella enterica* serovars Paratyphi (*S.* Paratyphi) A, B, and C, with Paratyphi A now being the predominant cause globally. According to the Global Burden of Disease Study 2017, an estimated 3.4 million cases of paratyphoid fever occurred worldwide in 2017 [1]. Although the case fatality rate is generally under 1% with appropriate treatment, complications such as intestinal perforation and septicemia may increase mortality in regions with limited healthcare access [1]. The disease is primarily transmitted through ingestion of contaminated food or water, and it remains endemic in areas with inadequate sanitation, including South and Southeast Asia, sub-Saharan Africa, and parts of Oceania [2–4]. Within these endemic regions, countries such as India, Nepal, Pakistan, and parts of China have seen *S.* Paratyphi A become a notable cause of enteric fever, sometimes exceeding the prevalence of *S.* Typhi [4–6]. In contrast, paratyphoid fever cases reported in high-income countries are predominantly travel-related, with most infections traced to travelers returning from endemic areas [3,7,8].

The increasing prevalence of antimicrobial-resistant *S.* Paratyphi A strains, especially those with reduced susceptibility to fluoroquinolones, presents a significant clinical concern [9]. While most isolates remain susceptible to first-line agents such as ampicillin, chloramphenicol, and cotrimoxazole, as well as third-generation cephalosporins and macrolides, nonsusceptibility to fluoroquinolones, including reduced susceptibility and resistance, is commonly observed and often linked to mutations in the *gyrA* gene, particularly at codon 83 (S83F and S83Y) [5,10]. Fluoroquinolones have been widely used as empirical treatment for enteric fever, including paratyphoid fever caused by *S.* Paratyphi A, due to their oral availability and broad tissue penetration [11]. However, increasing fluoroquinolone nonsusceptibility has raised concerns about treatment failure and the need for alternative agents [9,10,12]

Recent phylogenomic analyses, including whole-genome sequencing of outbreak isolates from India, Bangladesh, and China, have significantly enhanced our understanding of *S.* Paratyphi A transmission dynamics and population structure [ 2,5,6]. These studies have revealed localized outbreaks and suggested regional circulation of predominant genotypes, thereby contributing to the rising disease burden in South and Southeast Asia. To facilitate comparative genomic analysis, Tanmoy et al. developed Paratype, an SNP-based genotyping tool that enables standardized classification of *S.* Paratyphi A genotypes (paratypes) [13]. Using this system, studies in Bangladesh, India, Cambodia, and China have identified the predominance of globally distributed lineages demonstrating intranational genetic homogeneity but marked interregional diversity [5,10]. In parallel, HierCC, a cgMLST-based hierarchical clustering scheme implemented by EnteroBase, has been applied to define genetic relatedness among *Salmonella* strains at multiple resolution levels [14]. In a recent study, most *S.* Paratyphi A cases in the UK were associated with travel to South Asia, and isolates from the same country often belonged to the same HC5 cluster, highlighting HierCC’s utility in tracking travel-related transmission routes [3].

In Taiwan, paratyphoid fever is a notifiable disease with a historically low incidence, primarily imported from endemic regions [15]. However, a notable increase in locally acquired infections since 2022 has raised concerns about ongoing domestic transmission. To clarify the origin and transmission dynamics of these recent cases, we analyzed the genomic and antimicrobial resistance profiles of *S.* Paratyphi A isolates from both imported and domestic infections and compared them with international strains.

## Methods

### Bacterial isolates and epidemiological data

In Taiwan, paratyphoid fever is a notifiable disease. Hospitals are required to report confirmed cases to local health authorities and submit isolates to the Taiwan Centers for Disease Control (Taiwan CDC) for all culture-confirmed cases. Bacterial isolates from reported patients were obtained primarily through hospital-based microbiological diagnosis and submitted to the Taiwan CDC laboratory for further confirmation and subtyping. At the Taiwan CDC, isolates were re-identified using the Bruker MALDI Biotyper system (Bruker Daltonics GmbH & Co. KG, Bremen, Germany) and analyzed by pulsed-field gel electrophoresis (PFGE) to predict serotypes as part of routine surveillance protocols [16]. All isolates were identified as *S.* Paratyphi A and stored in 15% glycerol stocks at –75 to –80 °C in a deep freezer for future analysis. It should be noted that this system primarily captures moderate to severe cases who seek medical care at hospitals and undergo blood or stool culture. Asymptomatic infections or mild community-acquired cases that do not lead to clinical testing are unlikely to be captured in the surveillance system. This inherent limitation may introduce a degree of selection bias, favoring more clinically apparent cases, and is acknowledged as a constraint of the present study. Statistical data on reported paratyphoid cases from 1 January 2001 to 31 December 2024 were obtained from the Taiwan National Infectious Disease Statistics System (NIDSS; https://nidss.cdc.gov.tw/en/Home/Index). Demographic information, including sex, age, country of citizenship, country of residence, travel history, and year of onset, was retrieved from the Taiwan National Notifiable Disease Surveillance System with authorization from the Taiwan CDC (IRB113107#1). The demographic characteristics of 213 paratyphoid fever cases reported in Taiwan from 2001 to 2024 are summarized in S1 Table.

### Antimicrobial susceptibility testing (AST)

Of the 135 confirmed cases of *S.* Paratyphi A infection between 2007 and 2024, bacterial isolates were available for 88 cases (S2 Table). All 88 isolates underwent antimicrobial susceptibility testing and whole-genome sequencing. Isolates from earlier years (2001–2006) were unavailable due to the lack of a standardized strain archiving system during that time. Isolates were tested with the EUVSEC3 Sensititre MIC panel (TREK Diagnostic Systems Ltd., West Essex, England), which includes 15 antimicrobial agents selected according to the European Union protocol for AMR monitoring in *Salmonella* spp. (EFSA & ECDC, 2025). This panel is used in harmonized surveillance across EU Member States, allowing for direct comparison with antimicrobial resistance data reported in the EU. The selection of antimicrobials reflects their clinical importance and relevance for public health. The MIC breakpoints for *Enterobacterales,* based on the Clinical and Laboratory Standards Institute (CLSI) guideline [17], were used to interpret the antimicrobial susceptibility testing results for amikacin, ampicillin, azithromycin, cefotaxime, ceftazidime, chloramphenicol, ciprofloxacin, colistin, gentamicin, meropenem, nalidixic acid, sulfamethoxazole, tetracycline, and trimethoprim. For tigecycline, however, no interpretative criteria are provided by CLSI, and the European Committee on Antimicrobial Susceptibility Testing (EUCAST) has not established an epidemiological cutoff value (ECOFF) for *Salmonella*. Therefore, we adopted the resistance breakpoint of >0.5 mg/L for tigecycline as used in the EU surveillance system [18]. For ciprofloxacin, MIC values were interpreted using CLSI breakpoints, where isolates were considered susceptible for MIC < 0.125 mg/L, intermediate for MIC = 0.125–0.5 mg/L, and resistant for MIC ≥ 1 mg/L. In this study, the term “nonsusceptible” refers to isolates that were either intermediate or resistant to ciprofloxacin.

### Whole genome sequencing (WGS) and analysis

Genomic DNA was extracted using the DNeasy Blood & Tissue Kit (cat. #69506, Qiagen, Hilden, Germany) according to the manufacturer’s instructions. Sequencing libraries were prepared using the Illumina DNA Prep kit (cat. IL20018705, Illumina Inc., San Diego, CA, USA), which includes tagmentation of genomic DNA, bead-based cleanup, PCR amplification with adapters, and final purification. Sequencing was performed on the Illumina MiSeq platform (Illumina Inc.), and all isolates achieved a depth of coverage > 30X. Raw reads were processed using fastp (https://github.com/OpenGene/fastp) for quality filtering and adapter removal. Filtered reads were assembled de novo using SPAdes v3.15.3 [19]. Low-quality contigs were excluded based on length (<200 bp), read coverage (<2X), or homopolymer composition. The resulting assemblies were analyzed using AMRFinderPlus [20] to identify antimicrobial resistance determinants, SISTR (https://github.com/phac-nml/sistr_cmd) for in silico serotype prediction, and PlasmidFinder (http://www.genomicepidemiology.org) for identifying plasmid incompatibility types. Multilocus sequence types (STs) were assigned using the mlst tool (https://github.com/tseemann/mlst).

### Genotype assignment and phylogenetic analysis

Paratypes were assigned using the Paratype tool (https://github.com/CHRF-Genomics/Paratype/) [13], and HierCC cluster assignments were retrieved from EnteroBase (https://enterobase.warwick.ac.uk/species/index/senterica). Core genome SNPs were identified using ska.rust v0.3.7 (https://github.com/bacpop/ska.rust) [21], with assembled contigs of each isolate mapped to the *S.* Paratyphi A reference genome AKU_12601 (GenBank accession: FM200053) [22], which was isolated in 2002 from a patient in Karachi, Pakistan. After generating the core genome SNP alignment, we masked SNP positions corresponding to known repetitive and recombinogenic regions using BEDTools v2.30.0 [23] and the PARAREPEATSCamila_Kat_Maq_merged_nonoverlapping.txt file from the Holt Lab (https://github.com/katholt/typhoid). This step removed common hotspots of recombination and genomic instability. We then used Gubbins v3.3.1 (https://github.com/nickjcroucher/gubbins) to detect and filter any additional recombination regions not captured by the initial masking. The final recombination-filtered SNP alignment was used to infer a maximum-likelihood phylogenetic tree with RAxML, implemented within the Gubbins pipeline, using the GTRGAMMA nucleotide substitution model. No bootstrap replicates were performed. The tree was midpoint-rooted and visualized using Interactive Tree of Life (iTOL) v6 (https://itol.embl.de) [24].

To explore the genetic relatedness between *S.* Paratyphi A isolates from Taiwan and those from other countries, we compared our 88 isolates with publicly available genome sequences from Bangladesh [2,4], Cambodia [10,25], China [6], India [5,26,27], Indonesia [3], Myanmar [3], Nepal [4,28], and Pakistan [3]. Global isolate sequences were obtained from the NCBI database. Their accession numbers and associated metadata, including geographic origin, year of isolation, paratype, sequence type, and *gyrA* mutation status, are listed in S3 Table.

### Time-scaled phylogenetic analysis

To estimate the time to the most recent common ancestor (tMRCA) of *S.* Paratyphi A paratype 2.4 isolates associated with domestically acquired, outbreak-related paratyphoid fever cases in Taiwan, a Bayesian phylogenetic dating analysis was conducted using BactDating v1.1.4 [29]. The analysis was based on a core genome SNP alignment from which recombination regions had been filtered before tree construction.

## Results

### Epidemiology of paratyphoid fever

**Fig 1:**
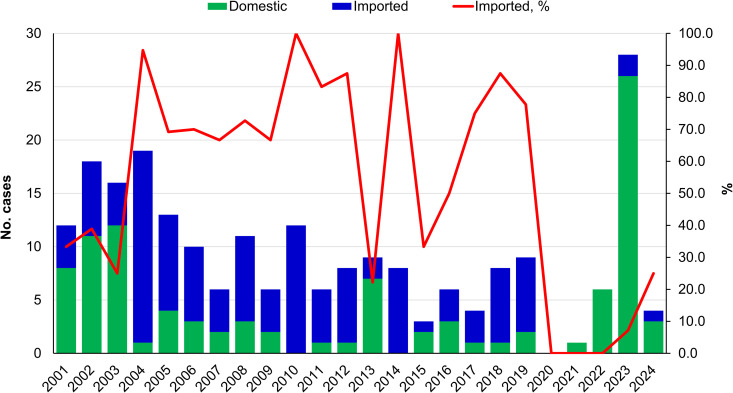
Epidemiology of Paratyphoid Fever in Taiwan. The data includes 223 confirmed cases of paratyphoid fever reported in Taiwan between 2001 and 2024. Case numbers reflect both imported and domestically acquired infections, as detailed in the Methods section.

Based on the NIDSS data described above, a total of 223 paratyphoid fever cases were reported in Taiwan between 2001 and 2024. During this period, 0–28 cases were reported annually, with an incidence ranging from 0 to 0.12 per 100,000 population. Among the 223 reported cases, 123 (55.2%) were classified as imported, and 100 (44.8%) as domestically acquired. As shown in Fig 1, imported cases accounted for the majority (>50%) of total cases in most years between 2004 and 2019, except for 2013 and 2015. After a near absence of cases during the COVID-19 pandemic (2020–2021) due to strict travel restrictions, an unexpected rise in locally acquired paratyphoid fever cases was observed between 2022 and 2024. Of the 123 imported cases, 40 (32.5%) originated from Indonesia, followed by China (27 cases, 22.0%) and India (22 cases, 17.9%), together accounting for 72.4% of all imported cases. Other countries contributing to imported cases include Cambodia (12 cases), Myanmar (8 cases), Bangladesh (4 cases), Nepal (2 cases), Thailand (2 cases), and five additional countries (5 cases). Imported cases comprised Taiwanese residents who acquired the infection during overseas travel, as well as foreign migrant workers and international travelers who were infected before entering Taiwan.

Demographic data for 213 of the 223 cases from 2001 to 2024 were summarized in S4 Table. Among the 213 cases, 117 (54.9%) were imported and 96 (45.1%) were acquired domestically. The cohort comprised 114 females (53.5%) and 99 males (46.5%). Statistical analyses showed no significant differences in sex distribution across all cases (p = 0.30), imported cases (p = 0.166), or domestically acquired cases (p = 1.0). The most common age groups were 25–29 years (15.0%), 30–34 years (13.1%), and 35–39 years (11.7%). A chi-square test showed a highly significant difference in the distribution of cases across age groups (p = 3.78 × 10 ⁻ ¹¹), with a disproportionately higher number of cases among young adults aged 25–39 years.

### Antimicrobial susceptibility

From 2007 to 2024, a total of 135 paratyphoid fever cases were confirmed in Taiwan, with isolates obtained from 88 cases (65.2%). AST revealed that all 88 isolates were susceptible to amikacin, gentamicin, azithromycin, ampicillin, third-generation cephalosporins (cefotaxime and ceftazidime), meropenem, colistin, chloramphenicol, sulfamethoxazole, trimethoprim, tetracycline, and tigecycline. However, 76.1% (67/88) of isolates were resistant to nalidixic acid and nonsusceptible to ciprofloxacin.

### Genomic characteristics and phylogenetic analysis of isolates from Taiwan

WGS was performed on 88 *S.* Paratyphi A isolates (51 imported and 37 domestic) obtained between 2007 and 2024. All isolates were confirmed as *S.* Paratyphi A by serotype prediction using the *Salmonella* In Silico Typing Resource (SISTR). Conventional sequence type analysis identified 26 isolates as ST85 and 62 as ST129 (S2 Table). PlasmidFinder identified plasmid replicons in a total of 14 isolates, including *Col(pHAD28)* (n = 11), *Col(BS512)* (n = 1), *Col(MG828)* (n = 1), and *IncX3(pEC14)* (n = 1). None of these plasmids carried known resistance genes in these isolates. Screening of antimicrobial resistance determinants using AMRFinderPlus did not find any known acquired resistance genes in the 88 isolates. The only resistance-associated mechanisms observed were chromosomal mutations in *gyrA*, which conferred resistance to nalidixic acid and reduced susceptibility to ciprofloxacin. Mutations in *gyrA* were detected in 67 ciprofloxacin-nonsusceptible isolates, with S83F substitution present in 64 isolates and S83Y in 3 isolates, while 21 isolates retained the wild-type *gyrA*. The isolates were classified into 11 paratypes, with the most common being paratype 2.4 (52 isolates), followed by 2.3.1 (8), 2.3.2 (7), and 2.3.3 (7) (S5 Table). Among the 37 domestic isolates, 34 were classified as paratype 2.4, which was also identified in 18 of the 19 isolates imported from Indonesia. The remaining three domestic isolates belonged to paratypes 2, 2.3.1, and 2.3.2, respectively. The isolates from Cambodia, China, and Myanmar were each assigned to a distinct paratype 2.3.1, 2.3.3, and 2.3.2, respectively; whereas the 13 isolates from India were distributed across five different paratypes.

**Fig 2:**
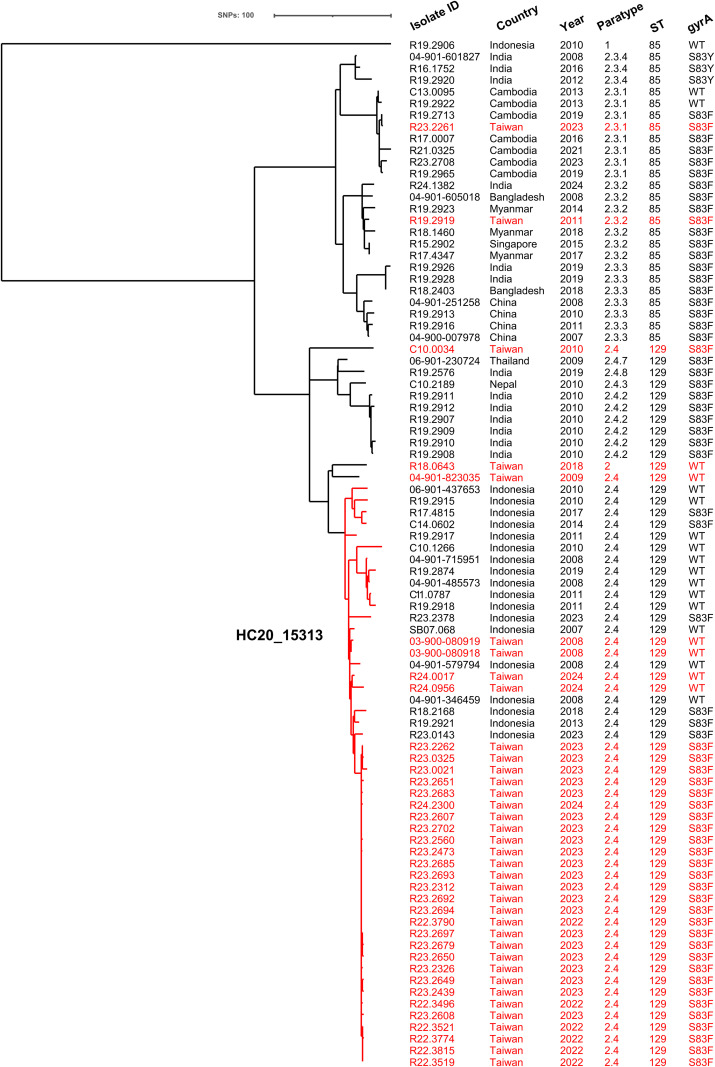
Phylogenetic tree of 88 *Salmonella enterica* serovar Paratyphi A isolates recovered in Taiwan between 2007 and 2024. The tree was inferred from core-genome single-nucleotide polymorphism (cgSNP) profiles using the maximum likelihood method. Isolates from domestically acquired cases are highlighted in red. The cluster containing 50 paratype 2.4 isolates from Taiwan and Indonesia is outlined in red and corresponds to HierCC cluster HC20_15313. Additional metadata for the 88 isolates are provided in S2 Table.

A phylogenetic tree based on the cgSNP profiles of 88 isolates displayed distinct clustering patterns corresponding to paratypes and geographic origins (Fig 2). Notably, 51 paratype 2.4 isolates and one paratype 2 isolate formed a distinct cluster comprising strains from both Indonesia and Taiwan, suggesting a potential epidemiological link between the two countries in the transmission of paratyphoid fever. Within this cluster, 50 paratype 2.4 isolates were assigned to HierCC HC20_15313, indicating that they differed by no more than 20 core genes in the cgMLST scheme. Among them, 28 domestic isolates collected between 2022 and 2024 shared key features, including paratype 2.4, sequence type ST129, and the *gyrA* S83F mutation. These 28 isolates differed by only 0–2 pairwise cgSNPs, all belonged to the same HC10 cluster (HC10_15313), and 27 were further grouped into HC5_506841 (S2 Table), reflecting a clonal group with near-identical genomic profiles. In contrast, pairwise SNP distances between paratype 2.4 isolates from different countries or years (e.g., early Indonesian isolates from 2007 to 2011 vs. recent Taiwanese outbreak strains) ranged from 8 to over 30 SNPs, indicating substantial substructure within this paratype. This high genomic similarity among the 28 recent domestic isolates supports the inference of a localized outbreak likely linked to a common external source. Of the remaining three domestic isolates detected during the same period, two (R24.0017 and R24.0956) also belonged to paratype 2.4 but harbored wild-type *gyrA* and were 8 SNPs away from the outbreak cluster. The third isolate (R23.2261) clustered with paratype 2.3.1 isolates from Cambodia.

### Spatiotemporal distribution of isolates from cases between 2007 and 2024

**Fig 3:**
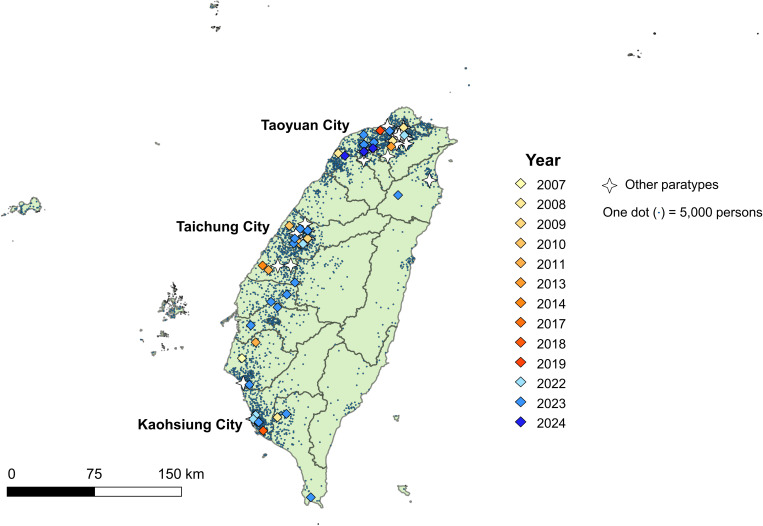
Spatiotemporal distribution of *Salmonella enterica* serovar Paratyphi A cases in Taiwan. Each case is mapped to the corresponding township or district of residence; however, for visual clarity, only city and county boundaries are displayed. Paratype 2.4 isolates from 2007 to 2019 are shown in shades of red, while those from 2022 to 2024, associated with the recent outbreak, are shown in shades of blue. Other paratypes are indicated by white star-shaped symbols. The background dot-density layer represents population distribution, with one dot corresponding to 5,000 persons. This figure was created using QGIS version 3.44x (https://qgis.org/). The basemap shapefile and demographic data were obtained from the Ministry of the Interior’s Statistical Map Service Platform (https://segis.moi.gov.tw/STATCloud/QueryInterface?DV=1) and are licensed under the Open Government Data License, version 1.0 (https://data.gov.tw/license).

To investigate the spatial and temporal distribution of *S. Paratyphi* A cases in Taiwan, all 88 isolates were mapped according to date of onset, paratype, and township or district of residence (Fig 3). These cases were primarily located in municipalities with higher population densities. Among the 32 paratype 2.4 cases identified between 2022 and 2024, most were geographically dispersed, with higher counts observed in Taoyuan City (n = 11), Taichung City (n = 5), and Kaohsiung City (n = 3); the remaining 13 cases were distributed across 9 other cities or counties. Notably, in Taoyuan City, Taichung City, and Kaohsiung City, several cases with proximate dates of onset resided in townships or districts that are geographically distant from one another. This broad spatial spread, even within the same municipality, suggests the absence of a tightly localized cluster.

### Genomic comparison of global isolates

**Fig 4:**
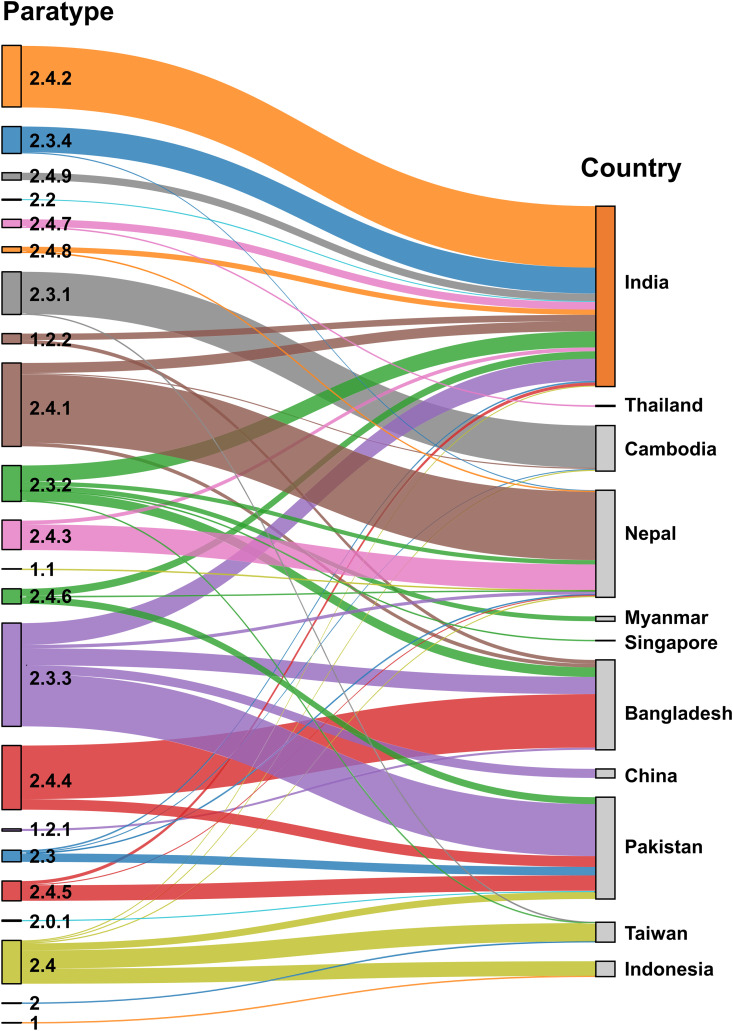
Sankey diagram showing the distribution of *Salmonella enterica* serovar Paratyphi A paratypes by country of origin. The diagram visualizes 1,074 isolates, with each flow representing the connection between a specific paratype and the reported country of origin. The width of each flow is proportional to the number of isolates, and each color corresponds to a distinct paratype.

To further examine the geographic origins of *S.* Paratyphi A isolates collected in Taiwan, we compared their paratype distribution with isolates from Bangladesh [2,4], Cambodia [10,25], China [6], India [5,26,27], Indonesia [3], Myanmar [3], Nepal [4,28], and Pakistan [3]. A Sankey diagram was constructed to visualize the relationships between countries of origin and paratypes (Fig 4). Among the 1,074 global isolates included (S3 Table), paratype 2.4.2 and 2.3.4 were predominantly found in India; 2.3.1 in Cambodia; 2.4.1 and 2.4.3 in Nepal; 2.3.2 in India and Bangladesh; 2.3.3 in India, Bangladesh, China, and Pakistan; 2.4.4 in Bangladesh and Pakistan; 2.4.5 in Pakistan; and 2.4 in Pakistan, Taiwan, and Indonesia. Isolates from India, Nepal, Pakistan, and Bangladesh exhibited high paratype diversity, while a single paratype dominated those from China, Taiwan, Indonesia, and Cambodia. Of the 37 domestic isolates, 34 (91.9%) were paratype 2.4, which is also common in Indonesia and has also been detected in several other South and Southeast Asian countries, including India, Nepal, Pakistan, and Cambodia.

To compare the results of paratype classification, we retrieved all HierCC assignments (HC0 to HC2850) for the isolates listed in S3 Table from EnteroBase. Of the 1,074 isolates, 786 had available HierCC designations. Among the various HierCC levels, the HC20 clusters showed the highest concordance with paratype classification. As shown in S6 Table, most paratypes corresponded to a single HC20 cluster. In contrast, paratypes 2.3, 2.3.2, 2.3.3, 2.4, and 2.4.5 were distributed across multiple clusters, reflecting intra-paratype genetic heterogeneity as revealed by cgMLST-based hierarchical clustering.

**Fig 5:**
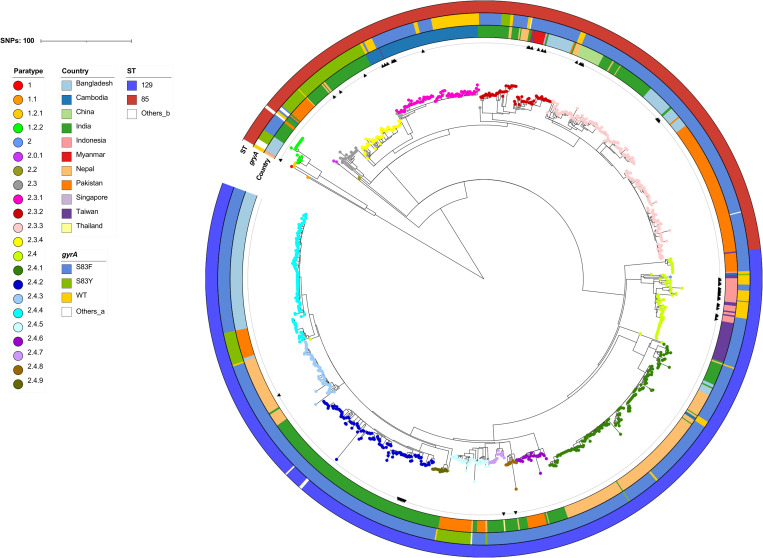
Phylogenetic tree of *Salmonella enterica* serovar Paratyphi A isolates. The tree was inferred from core genome single-nucleotide polymorphism (cgSNP) profiles using Gubbins v3.3.1 with maximum likelihood estimation, applying the General Time Reversible (GTR) substitution model and the Discrete Gamma correction. A total of 1,074 isolates from Taiwan and endemic countries were included. Colors indicate paratypes, countries of origin, and sequence types (STs), as shown in the accompanying legend. Arrows in the innermost ring denote isolates recovered in Taiwan from imported cases—that is, infections acquired abroad but diagnosed and cultured domestically. Others_a includes *gyrA* mutations D87Y, D87N, D87G, and the S83F-D87G double mutation; Others_b comprises ST1938, ST1939, and ST2216. Additional metadata are provided in S3 Table.

Phylogenetic analysis revealed that isolates within the same paratype generally exhibited a higher genetic relatedness (Fig 5). However, several paratypes, including 2.3.2, 2.3.3, 2.4, and 2.4.4, formed multiple distinct subclusters that were strongly associated with their geographic origins. For example, imported paratype 2.3.2 isolates from Bangladesh, Myanmar, and Singapore were located in a subcluster, separate from other subclusters containing isolates primarily from India and Nepal. Similarly, a subcluster of paratype 2.3.3 isolates from China, including four from imported cases, was distinct from other subclusters populated by isolates from Bangladesh, India, and Pakistan. The paratype 2.4 cluster, which also included one paratype 2 isolate, contained two major subclusters. Subcluster A consisted of isolates from Pakistan and one 2010 isolate (C10.0034) from Taiwan, while subcluster B primarily comprised isolates from Indonesia, Cambodia, and Taiwan (S1 Fig). Additionally, four paratype 2.4 isolates from Bangladesh and India were genetically distant from both subclusters. All domestic paratype 2.4 isolates identified between 2022 and 2024 showed high genetic similarity to Indonesian isolates. Notably, two 2024 isolates (R24.0017 and R24.0956), which retained the wild-type *gyrA* allele, were more genetically distinct from isolates carrying the *gyrA* S83F mutation, suggesting differences in their evolutionary trajectories or epidemiological origins.

### Time-scaled phylogeneny

To investigate the temporal origin of the recent outbreak-associated *S.* Paratyphi A isolates in Taiwan, a time-scaled phylogeny was inferred using BactDating based on core genome SNPs from 81 paratype 2.4 isolates originating from Taiwan, Indonesia, Cambodia, India, Nepal, and Pakistan. The analysis revealed that the majority of the Taiwanese isolates clustered closely with those from Indonesia, forming a monophyletic group with an estimated time to the most recent common ancestor (tMRCA) of 1935 (95% credible interval [CI], 1920–1945) (S2 Fig). Within this group, the 28 Taiwanese isolates associated with domestically acquired cases from 2022 to 2024 formed a distinct subcluster. The tMRCA for this subcluster was estimated to be 2015 (95% CI, 2011–2018), which could reflect a single-source introduction that occurred several years before the rise in reported domestic cases.

## Discussion

This study indicates a prolonged outbreak of paratyphoid fever in Taiwan between 2022 and 2024, during which domestically acquired cases markedly increased and outnumbered imported cases. Before 2022, most cases were imported, primarily from Southeast and South Asia, consistent with the trend observed in Taiwan between 1987 and 1996 [15]. The sudden predominance of domestic cases initially raised concerns about the emergence of sustained local transmission. However, genomic and epidemiological analyses indicate that these cases were more likely the result of repeated exposure to a common external source, such as contaminated imported food, rather than a fundamental shift in the epidemiological pattern of *S.* Paratyphi A infection in Taiwan.

The patterns shown in Fig 3 suggest that local cases identified since 2022 lacked clear temporal, geographic, or interpersonal links. A majority of affected individuals reported recent seafood consumption, supporting the hypothesis that environmental contamination, including that of seafood, may contribute to the transmission. Genomic analysis indicates that most domestic isolates have a close genetic relationship with Indonesian isolates, suggesting a potential epidemiological link between the two countries in the transmission of paratyphoid fever (S1 Fig). Among the 31 domestic isolates collected between 2022 and 2024, 30 clustered with Indonesian strains, and 28 exhibited high genetic similarity, sharing features such as paratype 2.4, ST129, and the *gyrA* S83F mutation. The strong genetic relatedness points to a common source of infection. In the absence of spatiotemporal clustering or direct epidemiological links among cases (Fig 3), the combined findings suggest repeated exposure to a shared food or environmental source, rather than ongoing local transmission or a single point-source outbreak. A similar pattern was observed during a paratyphoid fever outbreak in Mie Prefecture, Japan, in 1993, where consumption of raw oysters contaminated by *S.* Paratyphi A in harbor waters was identified as the probable source of infection [30]. Notably, the Japanese outbreak exhibited a pattern of geographically dispersed cases without person-to-person transmission, suggesting that environmental exposure played a central role and provided a relevant point of comparison for interpreting the current findings.

Among the 88 *S.* Paratyphi A isolates analyzed in this study, 67 (76.1%) carried mutations in *gyrA*, including 64 with the S83F substitution and 3 with the S83Y substitution. These mutations were associated with resistance to nalidixic acid and reduced susceptibility to ciprofloxacin. This finding is consistent with reports from Bangladesh, Cambodia, and India, where single-point mutations in *gyrA* at codons 83 or 87 are commonly linked to decreased fluoroquinolone susceptibility [2,5,10]. Although studies from China, India, and Japan have identified additional mutations within the quinolone resistance-determining region as well as plasmid-mediated resistance genes such as *aac(6’)-Ib-cr* [31–33], none were detected in our isolates. All isolates remained susceptible to the other 13 antimicrobials tested, including traditional first-line agents such as ampicillin, chloramphenicol, and trimethoprim-sulfamethoxazole, as well as third-generation cephalosporins, macrolides, aminoglycosides, carbapenems, and others. These results suggest that fluoroquinolone resistance, primarily driven by *gyrA* point mutations, remains the predominant type of antimicrobial resistance among *S.* Paratyphi A isolates collected in Taiwan.

In this study, we initially considered using all publicly available *S.* Paratyphi A genomic sequences from public databases, including NCBI, for comparison with our Taiwanese isolates. However, as noted by Chattaway et al. [3], we recognized that the “country” metadata in these databases often reflects the location where the genome was sequenced or uploaded, rather than the true geographic origin of infection. This limitation raises important concerns about the reliability of such data for global comparative analyses. To address this issue, we confined our comparative analysis to isolates from endemic regions with country-of-origin metadata verified by the original investigators. This strategy enabled more accurate geographic attribution and strengthened the validity of our global comparison.

Analysis of publicly available *S.* Paratyphi A genomes from NCBI reveals that the prevalent paratypes identified in our study are also frequently detected in isolates from high-income, non-endemic countries, including the United Kingdom, the United States, Japan, Canada, and Australia. However, consistent with the findings of Chattaway et al. [3], these isolates are likely derived from travel-associated cases rather than locally acquired infections. These observations reinforce the importance of accounting for potential metadata biases when conducting global comparative genomic analyses using data from publicly available databases such as NCBI.

While paratype 2.4 was predominant among both Taiwanese and Indonesian isolates, cgSNP-based phylogenetic analysis revealed considerable genetic divergence within this paratype. For example, paratype 2.4 isolates from Pakistan, India, and Nepal were not only genetically distant from those from Taiwan and Indonesia but also from each other (S2 Fig). Although isolates classified under the same paratype generally showed higher genetic similarity compared to those of different paratypes, several exceptions were noted. These findings suggest that the Paratype scheme may not provide sufficient resolution to distinguish between strains with distinct geographic origins. In contrast, cgSNP-based whole-genome comparisons offer a higher resolution, enabling differentiation between strains that belong to the same paratype but are genetically distinct.

This study has several limitations. First, our analysis was based exclusively on culture-confirmed isolates reported through the national surveillance system, which likely underrepresents milder or asymptomatic infections that were not diagnosed or reported, potentially introducing bias toward more severe cases. Second, the number of domestically acquired isolates collected between 2022 and 2024 was relatively small, limiting our ability to investigate the transmission dynamics or identify the outbreak source. Third, while genomic data indicated close relatedness to strains from Indonesia, the lack of detailed epidemiological information, such as food consumption histories or contact tracing, hindered the identification of potential infection sources. Although *S.* Paratyphi A is strictly human-adapted and does not reside in animal or environmental reservoirs, future studies incorporating food traceback investigations and targeted sampling in high-risk settings (e.g., food distribution chains) may help to clarify the transmission routes.

## Conclusion

Genomic analysis revealed that *S.* Paratyphi A isolates from recent domestically acquired infections in Taiwan are closely related to strains from Indonesia. Most domestic isolates recovered since 2022 are ciprofloxacin-nonsusceptible, belong to sequence type ST129 and paratype 2.4, and show high genetic similarity, suggesting repeated exposure to a common external source such as contaminated food or environmental reservoirs. These findings underscore the critical role of genomic surveillance in tracing the sources of infection and enabling rapid public health responses, particularly related to food and environmental safety. The continued susceptibility of these isolates to traditional first-line antibiotics supports their use in current treatment strategies.

## Supporting information

S1 Fig Phylogenetic tree of *Salmonella enterica* serovar Paratyphi A isolates. This figure includes all paratype 2.4 isolates (n = 81) and one paratype 2 isolate (IsolatID: R18.0643) in this study. The tree was inferred from core genome single-nucleotide polymorphism (cgSNP) profiles using maximum likelihood estimation. Isolates recovered in Taiwan are highlighted in red. Additional metadata for the 82 isolates are provided in S3 Table.
(TIF)

S2 Fig Time-scaled phylogeny of paratype 2.4 *Salmonella enterica* serovar Paratyphi A isolates. The tree includes 81 isolates from Taiwan, Indonesia, Cambodia, India, Nepal, and Pakistan, inferred using BactDating based on core genome SNPs.
(TIF)

S1 Table Demographic characteristics of 213 *Salmonella enterica* serovar Paratyphi A cases reported to the Taiwan CDC between 2001 and 2024. (XLSX)

S2 Table Detailed information on 88 *Salmonella enterica* serovar Paratyphi A isolates recovered from paratyphoid fever cases in Taiwan, 2007–2024. (XLSX)

S3 Table Detailed information on 1,074 *Salmonella enterica* serovar Paratyphi A isolates from various countries for phylogenetic comparison. (XLSX)

S4 Table Distribution of sex and age groups among 213 imported and domestically acquired paratyphoid fever cases in Taiwan, 2001–2024. (XLSX)

S5 Table Distribution of *Salmonella enterica* serovar Paratyphi A paratypes among isolates collected in Taiwan, 2007–2024. (XLSX)

S6 Table Distribution of HC20 clusters among *Salmonella enterica* serovar Paratyphi A isolates by paratype. (XLSX)
