# Correction: Paratyphoid fever and the genomics of *Salmonella enterica* serovar Paratyphi A in Taiwan

**DOI:** 10.1371/journal.pntd.0013673

**Published:** 2025-11-03

**Authors:** Ying-Shu Liao, Yu-Ping Hong, Bo-Han Chen, You-Wun Wang, Ru-Hsiou Teng, Shiu-Yun Liang, Hsiao Lun Wei, Jui-Hsien Chang, Ming-Hao Yang, Chi-Sen Tsao, Chien-Shun Chiou

The fourth author’s name is spelled incorrectly. The correct name is: You-Wun Wang. The correct citation is: Liao Y-S, Hong Y-P, Chen B-H, Wang Y-W, Teng R-H, Liang S-Y, et al. (2025) Paratyphoid fever and the genomics of *Salmonella enterica* serovar Paratyphi A in Taiwan. PLoS Negl Trop Dis 19(9): e0013048. https://doi.org/10.1371/journal.pntd.0013048.
